# Single-step genomic BLUP with many metafounders

**DOI:** 10.3389/fgene.2022.1012205

**Published:** 2022-11-21

**Authors:** Andrei A. Kudinov, Minna Koivula, Gert P. Aamand, Ismo Strandén, Esa A. Mäntysaari

**Affiliations:** ^1^ Natural Resources Institute Finland (Luke), Jokioinen, Finland; ^2^ Nordic Cattle Genetic Evaluation, Aarhus, Denmark

**Keywords:** genetic groups, genomic evaluation, red dairy cattle, finncattle, co-variance function

## Abstract

Single-step genomic BLUP (ssGBLUP) model for routine genomic prediction of breeding values is developed intensively for many dairy cattle populations. Compatibility between the genomic (**G**) and the pedigree (**A**) relationship matrices remains an important challenge required in ssGBLUP. The compatibility relates to the amount of missing pedigree information. There are two prevailing approaches to account for the incomplete pedigree information: unknown parent groups (UPG) and metafounders (MF). unknown parent groups have been used routinely in pedigree-based evaluations to account for the differences in genetic level between groups of animals with missing parents. The MF approach is an extension of the UPG approach. The MF approach defines MF which are related pseudo-individuals. The MF approach needs a **Γ** matrix of the size number of MF to describe relationships between MF. The UPG and MF can be the same. However, the challenge in the MF approach is the estimation of **Γ** having many MF, typically needed in dairy cattle. In our study, we present an approach to fit the same amount of MF as UPG in ssGBLUP with Woodbury matrix identity (ssGTBLUP). We used 305-day milk, protein, and fat yield data from the DFS (Denmark, Finland, Sweden) Red Dairy cattle population. The pedigree had more than 6 million animals of which 207,475 were genotyped. We constructed the preliminary gamma matrix (**Γ**_*pre*_) with 29 MF which was expanded to 148 MF by a covariance function (**Γ**_148_). The quality of the extrapolation of the **Γ**_*pre*_ matrix was studied by comparing average off-diagonal elements between breed groups. On average relationships among MF in $\Gamma_{148}$ were 1.8% higher than in **Γ**_*pre*_. The use of **Γ**_148_ increased the correlation between the **G** and **A** matrices by 0.13 and 0.11 for the diagonal and off-diagonal elements, respectively. [G]EBV were predicted using the ssGTBLUP and Pedigree-BLUP models with the MF and UPG. The prediction reliabilities were slightly higher for the ssGTBLUP model using MF than UPG. The ssGBLUP MF model showed less overprediction compared to other models.

## 1 Introduction

Genomic prediction in dairy cattle started in 2009 for US Holsteins, Jersey, and Brown Swiss (Wiggans et al., 2017). Since then, most dairy populations publish genomic estimated breeding values (GEBV) using a multi-step approach (Masuda et al., 2022). The term “multi” stands for a cascade of steps used to obtain GEBV: calculation of pseudo-observations for genotyped proven bulls and cows, estimation of SNP effects, prediction of direct genomic values, and blending of genomic values with pedigree index (Wiggans et al., 2011). In contrast, a single-step genomic BLUP (ssGBLUP) model accounts for pedigree, phenotypic, and genomic data simultaneously to obtain GEBVs for all animals (Legarra et al., 2009; Aguilar et al., 2010; Christensen and Lund, 2010). Despite the preselection bias in the multi-step GEBV (Patry and Ducrocq, 2011) and the benefits of the single-step model (Legarra et al., 2014), the latter is used only for a few dairy populations (Mäntysaari et al., 2020; Misztal et al., 2020; Masuda et al., 2022). High computational load, compatibility challenges for the genomic and the pedigree relationship matrices, and improper accounting of unknown parents impede the wide implementation of the single-step approach (Mäntysaari et al., 2020). In dairy cattle, these problems can be expected to be amplified due to the many generations in pedigrees, intensive selection, and the vast exchange of breeding material between populations.

The original ssGBLUP requires the inverse matrices of **A**_22_ and **G** in the inverted joint relationship matrix **H** ^−1^ (Aguilar et al., 2010; Christensen and Lund, 2010) where **A**_22_ is the pedigree relationship matrix of the genotyped animals and **G** is the genomic relationship matrix. When the number of genotyped animals in the **G** matrix (*n*) exceeds the number of markers (*m*), direct inversion of the **G** matrix is not possible without regularization such as adding a small value to the diagonal or a residual polygenic matrix (Mäntysaari et al., 2017). When n >> m, the single-step method becomes computationally challenging. Several computational approaches have been proposed for the computation of **G** ^−1^ to allow feasible application of ssGBLUP for large datasets (see review by Misztal et al., 2020). For instance, the method called ssGTBLUP (Mäntysaari et al., 2017) uses the relationship matrix of genotyped animals (**A**_22_) as the regularization matrix to avoid singularity, the Woodbury matrix identity for the **G** inverse, and a sparse presentation of the **A**_**22**_^−1^ to solve the computational challenges. In the data set with 178K genotyped animals to obtain GEBV, the ssGTBLUP model used 33% of the memory and 55% of the wall-clock time needed by the original ssGBLUP (Koivula et al., 2021a).

The difference in average off- and diagonal elements of **A**_22_ and **G** matrices is known as a single-step compatibility issue (Vitezica et al., 2011). To balance the matrices implies adjusting either the pedigree or the genomic relationship matrix to make the matrices more similar. The concept of the **A** adjustment was suggested by Christensen (2012) and further developed into the metafounder (MF) approach (Legarra et al., 2015). Metafounders are related inbreed pseudo-individuals that are used as unknown parents in the pedigree. Relationships between MF are described by a covariance matrix (**Γ**), which is used to build a relationship matrix **A** ^Γ^. Estimation of **Γ** can be based on estimates of base allele frequencies (AF) for each MF (Garcia-Baccino et al., 2017). An important assumption of the MF approach is that the **G** matrix is constructed with all AF equal to 0.5 (Legarra et al., 2015). Applicability of MF has been shown in livestock (Koivula et al., 2021b, 2022), sheep (Granado-Tajada et al., 2020), and pig (Xiang et al., 2017) data sets. The MF approach was also reported as a perfect choice for multi-breed evaluations in case computation of accurate **Γ** is possible (Poulsen et al., 2022).

The number of MF in the reported studies on ssGBLUP in the large dairy cattle breeds has nearly always been less than the number of unknown parent groups (UPG). Allocation of few MF by breed or by breed by time help to achieve an accurate estimation of **Γ** due to the even distribution of MF across genotyped animals (Kudinov et al., 2020; Masuda et al., 2021). When MF are used in ssGBLUP, it would be natural to use the same number of MF as there are UPG in the pedigree-based animal model (PBLUP). However, accurate estimation of an unstructured **Γ** matrix of large size is difficult, especially if some of the UPG groups have no descendants among the genotyped animals or genotyped individuals are several generations away.

The aim of this study was to propose an approach to construct **Γ** with the same number of MF as routinely defined UPG. The proposed approach was applied to the Red Dairy Cattle 305-day data and pedigree used for the milk production evaluation in Nordic countries (Denmark, Finland, and Sweden). Both PBLUP and ssGTBLUP models were used. The predictions used either UPG or MF in equal numbers. Thus, the predictive performance of four models was investigated.

## 2 Materials and methods

### 2.1 Data

Data were 305-day milk, protein, and fat yield records from three lactations of Nordic Red Dairy Cattle (RDC), Finnish Holstein (HOL), and Finncattle (FIC) cows. Records were from January 1988 to June 2021. The total number of records by trait were: 9.45, 8.99, and 8.98 million for milk, protein, and fat, respectively (Table 1). Pedigree included 6.05 million cows and 118,363 bulls, of which 8,427 were RDC and 278 were FIC proven bulls. Genetic groups were defined as breed x country x five- or 10-year period for RDC, and as breed x five- or 10-year period for HOL, FIC, and other breeds. In total, there were 148 groups: 61 RDC, 45 HOL, 16 FIC, and 26 for breed group OTHER. The group OTHER included 23 breeds majorly beef cattle.

**TABLE 1:** Number of records by lactation, trait, and breed in 305-day Nordic (Denmark, Finland, Sweden) Red Dairy cattle production data.

	RDC^a^			HOL^b^			FIC^c^		
Lactation	I	II	III	I	II	III	I	II	III
Milk	3,468,211	2,516,689	1,546,056	837,905	628,763	394,169	27,620	19,227	12,103
Protein	3,362,027	2,414,026	1,466,707	771,209	567,914	349,911	24,999	17,381	10,896
Fat	3,361,935	2,413,956	1,466,662	771,214	567,913	349,911	24,999	17,382	10,896

^a^ Red Dairy cattle.

^b^ Finnish Holstein.

^c^ Finncattle.

Genomic data were used from 206,140 RDC animals (6,018 proven bulls and 85,142 cows with records) and 1,335 FIC animals (160 proven bulls and 845 cows with records). Before 2019 the bulls were genotyped with Illumina Bovine SNP50 array and most cows with Illumina Bovine LD array (Illumina, San Diego, CA, USA). Since 2019 both bulls and cows were genotyped with Eurogenomics EG MD array (https://www.eurogenomics.com/). Quality control and imputation of genotypes to 46,914 SNPs were performed by NAV (Nordic Genetic Evaluation, Denmark). Genomic markers were not filtered on minor allele frequency and no edits were done concerning across and within breeds polymorphism. HOL genotypes were not presented in the current study.

### 2.2 Statistical models

Four prediction models were investigated using a multi-trait multi-lactation model: single-step GTBLUP with UPG in **H** ^−1^ (ssUPG), single-step GTBLUP with MF (ssMF), pedigree-based BLUP with UPG in **A** ^−1^ (pUPG), and pedigree-based BLUP with MF (pMF). The traits were milk, protein, and fat yield in three lactations i.e. - nine traits total. The linear mixed effects model was:

y=Xb+Zu+e,

where **y** is the vector of phenotypes, **X** is the design matrix relating fixed effects to the phenotypes, **b** is the vector of fixed effects, **Z** is the design matrix relating the breeding values to the phenotypes, $u∼N\left(0,A\sigma_{u}^{2}\right)$ is the vector of random animal breeding values, and $e∼N\left(0,I\sigma_{e}^{2}\right)$ is the residual vector. Matrix **A** is the pedigree-based relationship matrix, I is an identity matrix, $\sigma_{u}^{2}$ and $\sigma_{e}^{2}$ are genetic and residual variances, respectively. Fixed effects in **b** were calving year by season, calving age, herd by year, and calving age by breed. Calving age by breed effect consists of linear (α), quadratic (α^2^), and cubic (α^3^) regression coefficients of calving age multiplied by pedigree-based breed proportions of an animal (Lidauer et al., 2015) so that the general level of breed remained to be modeled by **u**. The regression coefficients were centered over all data to zero according to mean calving age as α=(calving age−calving age)/365 .

#### 2.2.1 Single-step GTBLUP

The mixed model equations (MME) of the original ssGBLUP model require the inverse of a joint relationship matrix **H** ^−1^ (Aguilar et al., 2010; Christensen and Lund, 2010):

$$H^{-1}={A\;}^{-1}+\left(\begin{array}{cc} 0 & 0\\ 0 & G^{-1}-A_{22}^{-1}\; \end{array}\right),$$

where, **A**_**22**_ is the part in **A** for the genotyped animals, and **G** is the genomic relationship matrix (VanRaden, 2008). Regularization matrix **C** = *w* **A**_22_ was added to the marker-based matrix **G**, where *w* is the residual polygenic proportion, i.e., the genomic relationship matrix was **G**_**c_w**_ = (1-*w*)**G**+ *w* **A**_22_ (Mäntysaari et al., 2017). We used *w* equal to 30% to keep the comparability to the studies by Koivula et al. (2021b, 2022). The **G**_**c_w**_ matrix was constructed with the assumption that AF of all markers was equal to 0.5. Thus, **G**_**c_w**_ = (1-*w*)**Z**_101_ **Z**_101_´/*k* + *w* **A**_22_ where *k* = *m*/2 is the scaling factor, *m* is the number markers, and **Z**_101_ is the matrix of genotype counts with values of 0 for the heterozygote and values -1 and +1 for homozygotes. The inverse genomic relationship matrix can be expressed as (Mäntysaari et al., 2017) $G_{c\_w}^{-1}=\frac{1}{w}A_{22}^{-1}-T_{w}^{\prime }T_{w}$ where $T_{w}=\frac{1}{w}L_{w}^{-1}Z_{101}^{\prime }\sqrt{\frac{2}{m}}A_{22}^{-1}$ and **L**_*w*_ is the Cholesky decomposition of $\frac{1}{w}Z_{101}^{\prime }A_{22}^{-1}Z_{101}\frac{2}{m}+\frac{1}{1-w}I$ .

#### 2.2.2 Single-step GTBLUP with UPG

The joint relationship matrix augmented by UPG (Quaas and Pollak, 1981; Misztal et al., 2013; Matilainen et al., 2018) was computed as shown in Koivula et al. (2021a):

$$H^{-1}=A_{UPG}^{-1}+\left(\begin{array}{ccc} 0 & 0 & 0\\ 0 & B_{11} & B_{12}\\ 0 & B_{21} & B_{22} \end{array}\right)$$

where

$$A_{UPG}^{-1}=\left(\begin{array}{ccc} A^{11} & A^{12} & -\left(A^{11}Q_{1}+A^{12}Q_{2}\right)\\ A^{21} & A^{22} & -\left(A^{21}Q_{1}+A^{22}Q_{2}\right)\\ -\left(Q_{1}^{\prime }A^{11}+{Q_{2}^{\prime }A}^{21}\right) & -\left(Q_{1}^{\prime }A^{12}+{Q_{2}^{\prime }A}^{22}\right) & Q^{\prime }A^{-1}Q \end{array}\right)$$

$$Q=\left(\begin{array}{c} Q_{1}\\ Q_{2} \end{array}\right),$$

and

$$B=\frac{1-w}{w}\;\left(\begin{array}{cc} A_{22}^{-1} & {-A}_{22}^{-1}Q_{2}\\ {-Q}_{2}^{\prime }A_{22}^{-1} & Q_{2}^{\prime }A_{22}^{-1}Q_{2} \end{array}\right)-\left(\begin{array}{cc} T_{w}^{\prime }T_{w} & {-T}_{w}^{\prime }T_{w}Q_{2}\\ -{Q_{2}^{\prime }T}_{w}^{\prime }T_{w} & {Q_{2}^{\prime }T}_{w}^{\prime }T_{w}Q_{2} \end{array}\right)$$

The **Q** matrix has proportions of genes contributed from each UPG according to pedigree information. The subscripts 1 and 2 in **Q** pertain to genotyped and non-genotyped animals. Subscripts 1 and 2 in **B** pertain to genotyped animals and UPGs, respectively. The UPGs were modeled as random effect. Inbreeding coefficients were accounted in both pedigree-based relationship matrices.

#### 2.2.3 Single-step GTBLUP with MF

In the MF approach (Christensen, 2012; Legarra et al., 2015), the **H** ^−1^ matrix was replaced by:

$${\left(H^{\Gamma }\right)}^{-1}={\left(A^{\Gamma }\right)}^{-1}+\left(\begin{array}{cc} 0 & 0\\ 0 & G_{c\_w}^{-1}-{\left(A_{22}^{\Gamma }\right)}^{-1} \end{array}\right),$$

where **G**_**c_w**_=(1-*w*)**G**+ *w* $A_{22}^{\Gamma }$ , **A** ^**Γ**^ is pedigree relationship matrix formed with a **Γ** matrix, $A_{22}^{\Gamma }$ is the submatrix of **A** ^**Γ**^ for the genotyped animals, and **Γ** was variance covariance matrix of the MF. Inbreeding coefficients estimated using the **Γ** matrix were used in the inverses of ${\left(A^{\Gamma }\right)}^{-1}$ and ${\left(A_{22}^{\Gamma }\right)}^{-1}$ .

#### 2.2.4 Pedigree BLUP

The pedigree-based models (pUPG and pMF) were similar to their corresponding single-step models, except that the genomic data was excluded from the prediction. In pUPG model UPGs were accounted in **A** ^−1^ ( $A_{UPG}^{-1}$ **;** Quaas and Pollak, 1981). In pMF model the **A** ^−1^ was replaced by ${\left(A^{\Gamma }\right)}^{-1}$ .

### 2.5 Estimation of the Γ matrix

Let the number of MF be *r* such that the **Γ** matrix has size *r*. In the MF approach, the **Γ** matrix describes the variance-covariance structure of MF. It can be estimated by 8Cov(P) , where **P** is an *m* by *r* matrix of estimated base population AF for each marker and MF (Legarra et al., 2015; Garcia-Baccino et al., 2017). In the studied data set, 44% of the UPG were not linked to the genotypes. Thus, using the 148 UPG as MF in the estimation of **P** was not feasible. To compute **Γ** for a large number of MF, the following general steps were used:

a) Estimate allele frequencies for a set of base groups;
b) From estimated allele frequencies, calculate the preliminary **Γ** matrix (**Γ**_pre_) for the base groups;
c) Solve the matrix **K** in the covariance function **Γ**_pre=_ ${Ф}_{pre}K{Ф}_{pre}^{\prime }$ + **E** using **Γ**_pre_ and the model matrix Φ_pre_; The matrix **E** is null if row rank of ${Ф}_{pre}$ is equal to dimension of **Γ**_pre_ and, if not **E** represents least squares errors of estimation.
d) Compute the **Γ** for the large number of groups as ${Ф}_{\Gamma }K{Ф}_{\Gamma }^{\prime }$ .

The model matrices ${Ф}_{pre}$ and ${Ф}_{\Gamma }$ define linear model by group and time for the set of base MF and for all MF, respectively.

**FIGURE 1:**
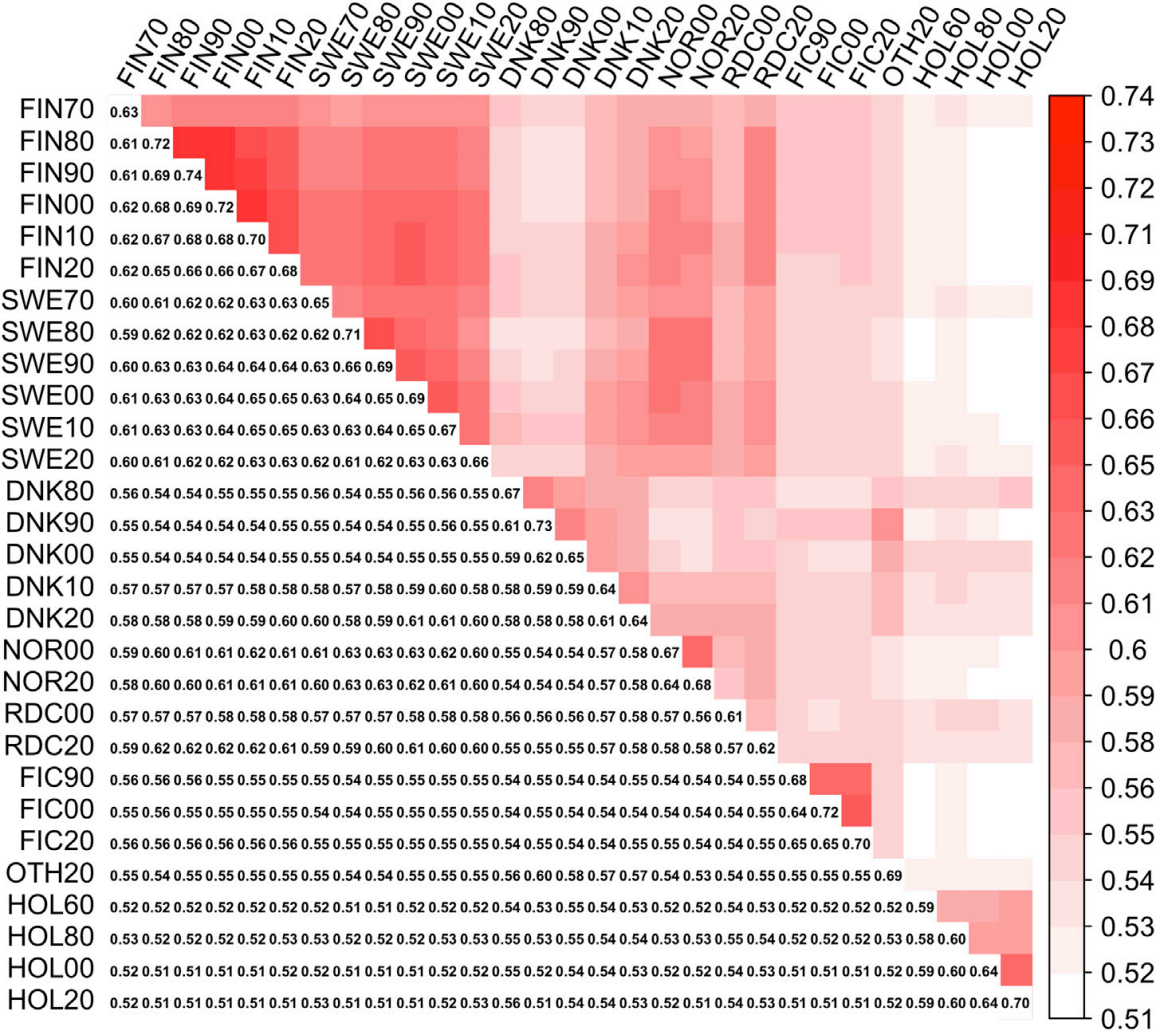
Symmetrical covariance matrix between 29 MFs (**Γ**_pre_). Lower triangle present diagonal (MFs self-relationships) and off-diagonal (between MFs relationships) elements in **Γ**_pre_ , upper triangle—heatmap plot of the off-diagonals.

The technical detailed steps used to compute **Γ** for 148 groups (**Γ**_148_) were:

a) The pedigree was pruned to include only one ancestor generation of genotyped animals as in Kudinov et al. (2020). Truncation of the pedigree helped to achieve equal distribution of the genomic information over UPGs. Missing parents in the truncated pedigree were replaced by 26 groups formed by breed, country, and time interval (Table 2). All HOL ancestors were assigned to the same group regardless of country and time. Estimation of base population AF for each of the groups (**P**_RDC_) was performed using the GLS method (McPeek et al., 2004; Garcia-Baccino et al., 2017). The HOL group estimated from RDC genotypes was dropped from **P**_RDC_. The HOL AF (**P**_HOL_) were the same as used in Kudinov et al. (2020)—calculated using Holstein genotypes (M. Koivula, personal communication). The joint **P**_RDC_HOL_ matrix of size 29 by 45,823 was created by merging compatible SNPs in **P**_RDC_ and **P**_HOL._ Number of SNPs dropped from **P**_RDC_ and **P**_HOL_ where 1,091 and 519, respectively.
b) Three **Γ** matrices (**Γ**_RDC_HOL_, **Γ**_RDC_, and **Γ**_HOL_) were computed using **P**_RDC_HOL_, **P**_RDC_, and **P**_HOL_. A *pre-* **Γ** matrix (**Γ**_pre_, Figure 1) was created by replacing the diagonal elements of **Γ**_RDC_HOL_ by diagonal elements of **Γ**_RDC_ and **Γ**_HOL_ at corresponding places. The diagonal values in **Γ**_pre_ were larger than in **Γ**_RDC_HOL_.
c) Structure of **Γ**_pre_ was computed with covariance function ${Ф}_{pre}K{Ф}_{pre}^{\prime }$ (Kirkpatrick et al., 1990), where Φ_pre_was a model matrix having standardized year of the MF (Appendix 1) and **K** was a matrix of co-variance function coefficients. Year standardization was done using formula $\frac{2\left({year}_{MF}-{year}_{\min}\right)}{{year}_{\max}-{year}_{\min}}-1$ , where *year*_MF_ is a year of the MF, *year*_min_ and *year*_max_ are the first (1950) and the last (2021) year points among the 148 groups in the pedigree.

**TABLE 2:** Groups used to compute preliminary **Γ** matrix.

Breed^a^	Origin^b^	Birth years of the animals descending from the group (MF)	Abbreviation	Genotype set^c^	Proportion of genotyped animals tied to the group (%)^d^
RDC	FIN	<1970	FIN70	RDC	3.62
RDC	FIN	1971–1980	FIN80	RDC	7.35
RDC	FIN	1981–1990	FIN90	RDC	7.91
RDC	FIN	1991–2000	FIN00	RDC	6.17
RDC	FIN	2001–2010	FIN10	RDC	7.29
RDC	FIN	2011–2020	FIN20	RDC	1.51
RDC	SWE	<1970	SWE70	RDC	4.20
RDC	SWE	1971–1980	SWE80	RDC	6.85
RDC	SWE	1981–1990	SWE90	RDC	11.00
RDC	SWE	1991–2000	SWE00	RDC	8.68
RDC	SWE	2001–2010	SWE10	RDC	1.14
RDC	SWE	2011–2020	SWE20	RDC	0.22
RDC	DNK	<1980	DNK80	RDC	2.17
RDC	DNK	1981–1990	DNK90	RDC	3.76
RDC	DNK	1991–2000	DNK00	RDC	4.26
RDC	DNK	2001–2010	DNK10	RDC	4.61
RDC	DNK	2011–2020	DNK20	RDC	0.80
RDC	NOR	<2000	NOR00	RDC	4.51
RDC	NOR	2001–2020	NOR20	RDC	0.24
RDC	ANY	<2000	RDC00	RDC	5.93
RDC	ANY	2001–2020	RDC20	RDC	1.20
FIC	FIN	<1990	FIC90	RDC	0.37
FIC	FIN	1991–2000	FIC00	RDC	0.15
FIC	FIN	2000–2020	FIC20	RDC	0.12
OTHER	ANY	<2020	OTH20	RDC	2.60
HOL	ANY	<1970	HOL70	HOL	13.42
HOL	ANY	1971–1990	HOL90	HOL	6.35
HOL	ANY	1991–2010	HOL10	HOL	3.06
HOL	ANY	2010–2020	HOL20	HOL	1.41

^a^ Breeds were RDC, Read Dairy Cattle; FIC, Finncattle; HOL, Holstein, and OTHER, other than listed.

^b^ Countries of origin: FIN, Finland; SWE, Sweden; DNK, Denmark; NOR, Norway, and ANY, any than specially specified.

^c^ RDC, and HOL, genotypes set include 46,914 and 46,342 markers, respectively. RDC, set included FIC, genotypes imputed along with RDC, genotypes.

^d^ Proportion of genotypes tied to the group in a particular genotype set.

Matrix **K** was estimated as (Tijani et al., 1999):

$${\hat{K}=\left({Ф}_{pre}^{\prime }{Ф}_{pre}\right)}^{-1}{{Ф}_{pre}^{\prime }\Gamma_{pre}Ф}_{pre}\;{\left({Ф}_{pre}^{\prime }{Ф}_{pre}\right)}^{-1}$$

leading to estimate

$$\hat{K}=\left[\begin{array}{ccccccccc} 0.0108 & 0.0093 & 0.0083 & -0.0042 & -0.0004 & 0.0003 & -0.0058 & -0.0054 & 0.0046\\ & 0.6563 & 0.6203 & 0.5551 & 0.5961 & 0.5850 & 0.5511 & 0.5434 & 0.5158\\ & & 0.6349 & 0.5621 & 0.6094 & 0.5795 & 0.5434 & 0.5415 & 0.5164\\ & & & 0.6098 & 0.5544 & 0.5644 & 0.5467 & 0.5778 & 0.5358\\ & & & & 0.6535 & 0.5678 & 0.5441 & 0.5375 & 0.5159\\ & & & & & 0.5899 & 0.5442 & 0.5458 & 0.5325\\ & & sym & & & & 0.6666 & 0.5530 & 0.5121\\ & & & & & & & 0.6946 & 0.5205\\ & & & & & & & & 0.6053 \end{array}\right]$$

d) Finally, the **Γ**_148_ was estimated as ${Ф}_{148}K{Ф}_{148}^{\prime }$ , where the matrix Φ_148_ was designed same way as Φ_pre_ but using all the MF. Rank of the **Γ**_148_ matrix is only 9. To avoid **A** ^**Γ**^ matrix singularity we reduced the off-diagonal values of **Γ**_148_ by 2.5% and increased the diagonal values by 2.5%

### 2.6 Validation of model fit

Validation of the prediction models was done using modified forward prediction (Mäntysaari et al., 2010). For the validation a reduced phenotypic data set was constructed by removing records from the last 4 years of data, i.e., June 2017 to June 2021. Daughter yield deviations (DYD) for bulls and yield deviations (YD) for cows were computed using the full data set using the same model which was applied to reduced data. Bias of evaluation was estimated by the linear regression coefficient (*b*_1_) from the weighted regression of DYD/YD on the corresponding [G]EBV predicted with the reduced data. The weight of DYD for bull *i* was EDC_*i*_/(EDC_*i*_ + λ_b_), where λ_b_ is (4—h^2^)/h^2^, h^2^ is heritability of the trait, and EDC_*i*_ is the effective daughter contributions of bull *i* computed as in Taskinen et al. (2014). Weight for cow YD_*j*_ was computed as ERC_*j*_/(ERC_*j*_ + λ_c_), where λ_c_ is (1-h^2^)/h^2^ and ERC_*j*_ is the effective record contribution of cow *j* (Přibyl et al., 2013). Adjusted validation reliability was attained by dividing the coefficient of determination from the regression model ( $R^{2}$ ) by the average weight of DYD ( $R_{EDC}^{2}$ ) and YD ( $R_{ERC}^{2}$ ) for bulls and cows, respectively. Average genetic trends were plotted using the trait specific combined [G]EBVs computed as

$${\left[G\right]EBV}_{parity\;1}*0.30+{\left[G\right]EBV}_{parity\;2}*0.25+{\left[G\right]EBV}_{parity\;3}*0.45$$

(https://nordicebv.info/wp-content/uploads/2021/10/NAV-routine-genetic-evaluation_EDITYSS-08102021.pdf).

### 2.7 Software

Pedigree truncation and estimation of the inbreeding coefficients was done using RelaX2 v.1.95 software. The AF were estimated using Bpop v. 0.98 program (Strandén and Vuori, 2006; Strandén and Mäntysaari, 2020), **T** matrix and its diagonal needed in the ssGTBLUP model were computed using hgtinv v.0.83 program. The computation of [G]EBV predictions and the estimation of EDC/ERC used MiX99 software (Strandén and Lidauer, 1999). MiX99 software uses preconditioned conjugate gradient (PCG) iteration. The PCG method was assumed to be converged when convergency criteria <1e-6 was achieved. Convergency criteria was defined as a Euclidean norm of the difference between the right-hand side (RHS) of the MME and the one predicted by the current solutions relative to the norm of RHS. The matrices ${\left(A_{22}\right)}^{-1}$ and ${\left(A_{22}^{\Gamma }\right)}^{-1}$ used by MiX99 and hginv were constructed using the given pedigree and inbreeding files, and in case of MF, by file with the **Γ** ^−1^ matrix.

## 3 Results and discussion

### 3.1 Relationship matrices

**FIGURE 2:**
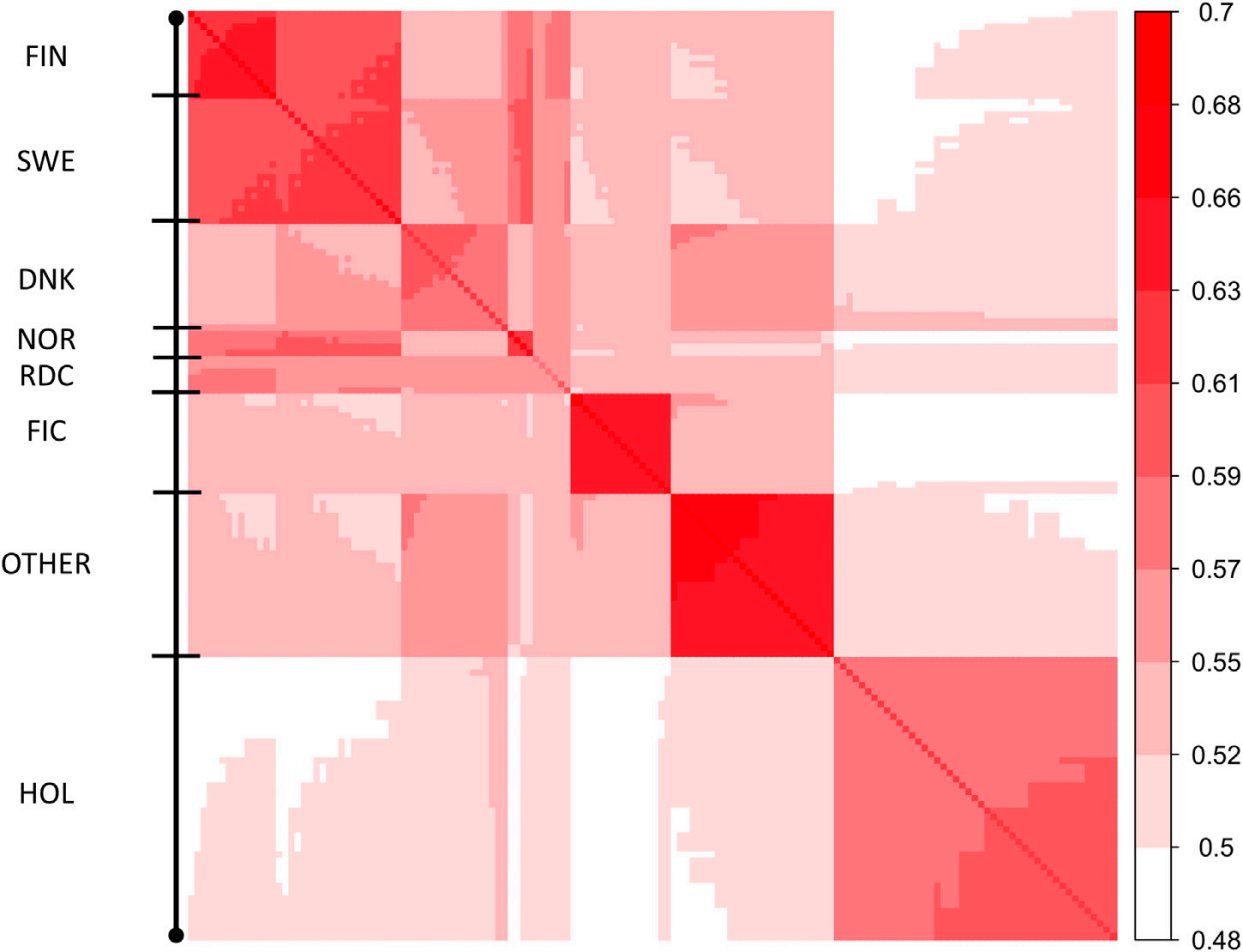
Heatmap of covariances between 148 MFs (**Γ**_148_). Diagonal of the heatmap plot are self-relationships of the MFs; off-diagonals are relationships between MFs.

Elements of **Γ**_*pre*_ ranged from 0.59 to 0.74 and from 0.51 to 0.69 for the diagonal and off-diagonal elements, respectively. The lowest and highest diagonal values (self-relationship, Legarra et al., 2015) were in groups HOL 1960 and RDC FIN 1990, respectively. In the **Γ**_148_ matrix, diagonal elements were in a range from 0.61 to 0.73 (Figure 2). The lowest and highest self-relationships were in HOL SWE 1970 and OTHER 1960 groups, respectively. The off-diagonal elements of **Γ**_148_ ranged from 0.48 to 0.69. The highest average relationships were observed between the FIN and SWE RDC groups, as expected. Relationships between HOL and RDC DNK were higher than with the other RDC groups due to the larger proportion of HOL sires in the RDC DNK pedigree. Similarly, the FIC groups were genetically closer to RDC FIN than to the other groups due to historical crossbreeding. Relationship coefficients between the RDC subgroups in our study ranged from 0.54 to 0.65 which was much higher than the range 0.09–0.18 presented between the biological types of Montana cattle breed (Kluska et al., 2021). Average relationships between RDC and HOL breed (0.52) was close to presented between HOL and Jersey breeds (0.48, Legarra et al., 2015).

Because **Γ**_148_ is an extrapolated matrix of **Γ**_*pre*_ we expect these to be alike. The difference between the two matrices was assessed using percentage deviation from the mean off-diagonal values in breed groups (Table 3). The average off-diagonal value of $\Gamma_{148}$ was 1.8% higher than in **Γ**_*pre*_. For instance, the average relationships between the RDC FIN and FIC groups were 0.54 and 0.56 in **Γ**_*pre*_ and **Γ**_148_, respectively. Thus, the covariance function allows to extrapolate the **Γ** matrix for the MF approach in order to have the same number of MF as UPG.

**TABLE 3:** Deviation from average relationships between breed groups in **Γ**_pre_ (lower triangle) and **Γ**_148_ (upper triangle).

	FIN	SWE	DNK	NOR	RDC	FIC	OTHER	HOL
FIN^a^		112.6^b^	99.7	107.5	105.2	99.1	97.8	93.1
SWE	110.1^b^		100.8	109.6	104.2	97.8	97.4	93.2
DNK	98.4	99.5		99.9	101.8	98.7	104.2	96.3
NOR	106.4	108.6	97.6		102.3	98.0	96.8	92.9
RDC	104.4	103.3	99.4	100.8		98.2	98.5	95.7
FIC	97.8	96.3	95.8	95.7	95.8		99.7	92.0
OTHER	96.3	95.9	101.3	94.5	96.0	96.8		93.6
HOL	91.3	91.4	94.7	91.6	94.5	90.7	92.0	

^b^ Deviation was computed as $\frac{\bar{\Gamma_{k,l}}}{\bar{\Gamma_{i,j\in i\neq j}}}*100$ , where **Γ**_*k,l*_ is submatrix of **Γ** for breed groups *k* and *l*, and $\Gamma_{i,j\in i\neq j}$ is off-diagonal submatrix of **Γ**

^a^ Groups were: FIN, Finnish Red Dairy Cattle; SWE, Swedish Red Dairy Cattle; DNK, Danish Red Dairy Cattle; NOR, Norwegian Red Dairy Cattle; RDC, Red Dairy Cattle from other countries; FIC, Finncattle; HOL, Finnish Holstein, and OTHER, breeds.

**FIGURE 3:**
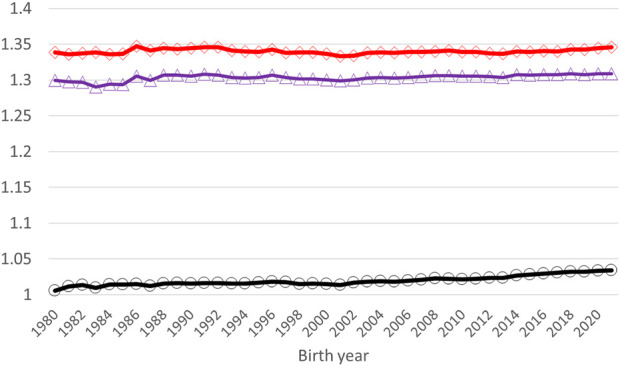
Average diagonal elements of **A**_22_ (black circles), **A** ^**Γ**^ (blue triangles), and **G**_**05**_ (red diamonds) by the birth year of a genotyped animal.

Application of the **Γ**_148_ matrix to the pedigree-based relationship matrix lifted the average diagonal elements of **A**_22_ closer to **G**_05_ (Figure 3). The smallest diagonal and off-diagonal values of **A**_22_ increased by 0.25 (from one to 1.25) and by 0.50 (from 0 to 0.50), respectively, by using $\Gamma_{148}$ as the basis for $A_{22}^{\Gamma }$ (Table 4). The increase was close to that in Kudinov et al. (2020) - 0.27 and 0.48 for the diagonal and off-diagonal elements, respectively**.** The correlations in the diagonal and off-diagonal elements were higher between **G**_05_ and $A_{22}^{\Gamma }$ (0.70 and 0.88) than between **G**_05_ and **A**_22_ (0.57 and 0.77). The overall magnitude of values in $A_{22}^{\Gamma }$ in our study was higher than presented for HOL in Koivula et al. (2022). Average relationship coefficients of **A**_22_, $A_{22}^{\Gamma }$ , and **G**_05_ in Koivula et al. (2022) had a steady increase by animal’s birth year. However, similar behavior in our study was observed only for the **A**_22_ matrix. A slight decrease in the average relationship coefficient of **G**_05_ and $A_{22}^{\Gamma }$ was observed after year 2000. This might be caused by the establishment of the joint Nordic RDC evaluation and admixture of the breads in the three populations. The total increase in the average relationships in the 40-year period were 2.81%, 0.97%, and 0.72% for **A**_22_, $A_{22}^{\Gamma }$ , and **G**_05_, respectively. The MF approach is beneficial in an admixed population such as Nordic RDC, as it helps to balance the **G**_05_ and **A**_22_ matrices. However, $A_{22}^{\Gamma }$ and **G**_05_ were not on exactly on the same scale as the mean diagonal and off-diagonal elements in $A_{22}^{\Gamma }$ were still somewhat lower than in **G**_05_ (Table 4). So, some compatibility issues between the pedigree- and genomic-based relationship matrices remained.

**TABLE 4:** Mean, minimum (Min), and maximum (Max) element values of **A**_22_, $A_{22}^{\Gamma }$ and **G**_**05**_ from diagonal and off-diagonal.

Elements	Matrix	Mean	Min	Max
Diagonal	**A**_22_	1.03	1.00	1.29
	$A_{22}^{\Gamma }$	1.31	1.25	1.50
	**G**_**05**_	1.34	1.00	1.58
Off-diagonal	**A**_22_	0.06	0.00	0.82
	$A_{22}^{\Gamma }$	0.61	0.50	1.17
	**G**_**05**_	0.68	0.47	1.40

**A**_22_ - the pedigree relationship matrix of genotyped animals **A**_22_^Γ^ - the pedigree relationship matrix of genotyped animals augmented by the **G**_**05**_ - the genomic relationship matrix with allele frequencies equal to 0.5.

In addition to **Γ**_*pre*_, **Γ**_RDC_HOL_ was tested as source for **Γ**_148*_ and corresponding $A_{22}^{\Gamma *}$ estimation. The mean difference between **Γ**_RDC_HOL_ and **Γ**_*pre*_ was 0.03. Diagonal elements in $A_{22}^{\Gamma *}$ constructed using extrapolated **Γ**_RDC_HOL_ were on average 0.02 lower than in $A_{22}^{\Gamma }$ used for genomic prediction. Even though construction of **Γ**_*pre*_ with **Γ**_RDC_ and **Γ**_HOL_ diagonals helped to lift **A**_22_ closer to **G**_05_, this step was not vital and **Γ**_RDC_HOL_ might have been used as it is.

Filtering of the SNPs by minor allele frequency (MAF) for the Γ matrix estimation was elaborated in our previous study (Kudinov et al., 2020), and indirectly performed in Legarra et al. (2015). In the current study, we avoided MAF filtering of SNPs used to compute $\Gamma_{pre}$ . That helped to compute a $A_{22}^{\Gamma }$ matrix closer to **G**_05_, as the same set of markers was used to construct **G**_05_. We observed that if selection of SNPs is used, it should be applied to both Γ and **G**_05_, i.e., the same set of markers should be used consistently. It is reasonable to keep the set of markers used in **G**_05_ and Γ as compatible as possible.

The presented approach in our study allows to fit the same number of MF as UPG and define MF for base population groups not linked to the genotypes. However, approach requires several arbitrary steps that need to be customized for each population. For instance, definition of the groups will be different in **Γ**_*pre*_. We defined the groups in **Γ**_*pre*_ by breed, country, and time. If any of defined groups had less than 0.1% of genotyped animals, we have had to combine it. In our study, the definition of the time variable in the base populations used to compute AF was the last year of the time interval, another way is to use mean, median or the first year. Because the year definition in each of the groups is used in the model matrix **Φ** and resulting covariance function, average diagonal of $A_{22}^{\Gamma }$ would expectedly decrease. The standardized year of the MF in **Φ** was computed with the same formulae. However, this can be adjusted for specific breed or country. Use of a covariance function in routine genomic prediction need re-estimation of the **Γ** matrix when new genetic groups are defined, but not re-estimation of base AF.

### 3.2 Model runs and validation

The ssMF and ssUPG models converged in 1,388 and 2,802 iterations. The wall-clock time per iteration was similar for ssMF and ssUPG models. The mix99 runtime for ssMF model in Intel Xeon 2.8 Ghz machine with four cores was 11 h 19 min.

Table 5 presents bull validation results for the nine traits. For all traits, the highest prediction reliability was obtained by the ssMF model. The regression slopes ( $b_{1}$ ) obtained by ssMF were slightly higher than by ssUPG. Prediction reliability by the pUPG and pMF models were the same. However, the slopes ( $b_{1}$ ) with pMF were closer to one than with pUPG. In all traits and models, quality of prediction decreased from lactation one to 3. For the validation cows (Table 6), the ssMF model gave slightly better validation reliability in milk than the ssUPG model. For protein and fat, the same prediction reliability was estimated in both single-step models. The slope in ssMF was closer to one than in the other models. However, in the first parity milk trait, the slope was slightly above one in ssUPG and ssMF. The MF approach improved quality of genomic prediction in the studied population similarly as reported in Bradford et al. (2019), Masuda et al. (2021), and Koivula et al. (2022) The bias of prediction in the ssMF model was lower as reported for dairy sheep (Macedo et al., 2020). Nonetheless bias in our study remain significant in all single-step models.

**TABLE 5:** [G]EBV validation test regression coefficients (*b*_1_) and weighted validation reliabilities ( $R_{EDC}^{2}$ ) for RDC validation bulls.

Model^a^		ssUPG		ssMF		pUPG		pMF	
Trait - parity	Number of bulls	*b*_1_ ^b^	$R_{EDC}^{2}$ ^c^	*b*_1_	$R_{EDC}^{2}$	*b*_1_	$R_{EDC}^{2}$	*b*_1_	$R_{EDC}^{2}$
Milk 1	284	0.80	0.45	0.82	0.48	0.82	0.20	0.87	0.21
Milk 2	243	0.77	0.37	0.81	0.39	0.89	0.22	0.93	0.22
Milk 3	180	0.76	0.21	0.79	0.22	0.91	0.13	0.96	0.13
Protein 1	287	0.64	0.34	0.66	0.36	0.54	0.11	0.59	0.11
Protein 2	240	0.62	0.30	0.70	0.31	0.66	0.14	0.70	0.14
Protein 3	181	0.66	0.18	0.68	0.19	0.74	0.10	0.80	0.10
Fat 1	284	0.68	0.40	0.71	0.41	0.63	0.17	0.67	0.17
Fat 2	241	0.73	0.39	0.78	0.40	0.70	0.16	0.73	0.16
Fat 3	181	0.72	0.21	0.75	0.20	0.79	0.12	0.83	0.12

^a^ Models are: ssUPG, single-step GTBLUP, with UPG, accounted in **H** ^−1^, ssMF, single-step GTBLUP, with MF; pUPG-pedigree BLUP, with UPG, accounted in **A** ^−1^, and pMF, pedigree BLUP, with MF.

^b^ Regression coefficient $b_{1}$ ^b^ in $DYD=b_{0}+b_{1}*\left[G\right]EBV$ equation has been multiplied by two.

^c^ Validation reliability was obtained from coefficient of determination of the model (R_model_^2^), after correcting it by the average weight of DYDs ( $R_{EDC}^{2}$ ).

**TABLE 6:** [G]EBV validation test regression coefficients (*b*_1_) and weighted validation reliabilities ( $R_{ERC}^{2}$ ) for RDC validation cows.

Model^a^		ssUPG		ssMF		pUPG		pMF	
Trait - parity	Number of cows	*b*_1_	$R_{ERC}^{2}$ ^b^	*b*_1_	$R_{ERC}^{2}$	*b*_1_	$R_{ERC}^{2}$	*b*_1_	$R_{ERC}^{2}$
Milk 1	32,133	1.03	0.50	1.04	0.51	0.91	0.17	0.97	0.16
Milk 2	27,097	0.96	0.41	0.97	0.42	0.93	0.15	0.98	0.15
Milk 3	10,923	0.91	0.34	0.92	0.34	0.85	0.13	0.90	0.13
Protein 1	33,298	0.87	0.36	0.89	0.36	0.80	0.14	0.84	0.14
Protein 2	25,491	0.82	0.33	0.84	0.33	0.83	0.13	0.88	0.13
Protein 3	10,387	0.83	0.29	0.84	0.29	0.78	0.11	0.83	0.11
Fat 1	34,129	0.88	0.38	0.90	0.38	0.84	0.15	0.89	0.15
Fat 2	25,987	0.83	0.32	0.85	0.32	0.85	0.14	0.90	0.14
Fat 3	10,494	0.86	0.31	0.86	0.31	0.82	0.13	0.87	0.13

^a^ Models are: ssUPG, single-step GTBLUP, with UPG, accounted in **H** ^−1^, ssMF, single-step GTBLUP, with MF; pUPG-pedigree BLUP, with UPG, accounted in **A** ^−1^, and pMF, pedigree BLUP, with MF.

^b^ Validation reliability was obtained from coefficient of determination of the model (R_model_^2^), after correcting it by the average weight of DYDs ( $R_{EDC}^{2}$ ).

**FIGURE 4:**
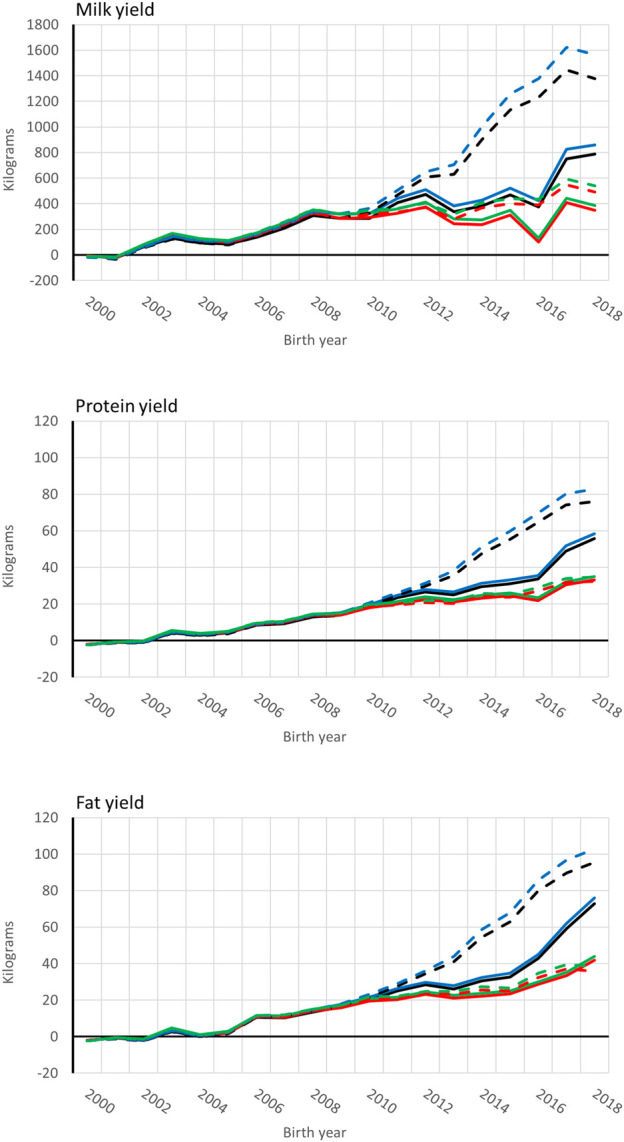
Average [genomic] breeding value of bulls by birth year in 305-d milk, protein, and fat yield (kg). Each bull had at least 50 daughters. Solid and dashed lines are from the model runs with full and reduced (minus four production year) data. Models are ssUPG—single-step GTBLUP with UPG accounted in **H** ^−1^ (blue lines), ssMF—single-step GTBLUP with MF (black lines); pUPG-pedigree BLUP with UPG accounted in **A** ^−1^ (green lines), and pMF—pedigree BLUP with MF (red lines).

**FIGURE 5:**
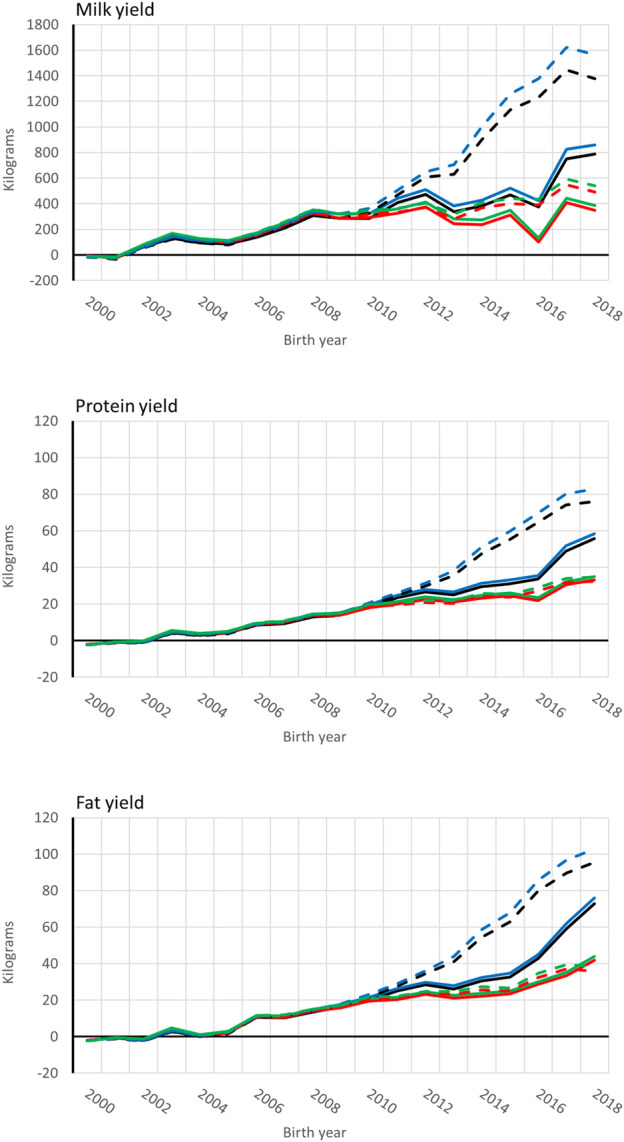
Average [genomic] breeding value of cows by birth year in 305-d milk, protein, and fat yield (kg). Solid and dashed lines are from the model runs with full and reduced (minus four production year) data. Models are ssUPG—single-step GTBLUP with UPG accounted in **H** ^−1^ (blue lines), ssMF—single-step GTBLUP with MF (black lines); pUPG-pedigree BLUP with UPG accounted in **A** ^−1^ (green lines), and pMF—pedigree BLUP with MF (red lines).

Genetic trends for combined milk, fat, and protein GEBVs are presented for the genotyped bulls with at least 50 daughters and all RDC cows in Figures 4, 5, respectively. Average GEBV were centered using the mean GEBV of RDC cows born in 2007. Both genomic and non-genomic models had similar shape in the UPG and MF instance. The average GEBV levels were higher than the average EBV levels. Similar difference has been observed in other single-step studies (Ma et al., 2015; Silva et al., 2019; Koivula et al., 2022). Overprediction in single-step with MF was reduced in our study similar to reported in Masuda et al. (2021) and Koivula et al. (2022).

Reduction of heritability by additive variance scaling was suggested by Legarra et al. (2015) when MF are used for genomic prediction. Base populations in models with UPGs are assumed unrelated, which is contrary to MF. In order to solve that problem additive variance was suggested to be scaled by $\left(1+tr\left(\Gamma \right)/\left(2n\right)-1^{\prime }\Gamma 1/n^{2}\right)$ , where tr(**Γ**) is the sum of diagonal elements of the **Γ** matrix (Legarra et al., 2015). However, this is based on assumption that the current population is a homogenous mixture of all the base populations that the MF will present. In reality base populations have influence unequally to the studied population, and thus we kept the same genetic variances in the UPG and MF models.

## 4 Conclusion

We presented a method to utilize the same number of MF as UPG in single-step GBLUP. The Covariance functions allowed smooth extrapolation of the Γ matrix with 29 metafounders to 148 in the pedigree of all animals. Use of $\Gamma_{148}$ increased correlation between the elements of pedigree and genomic relationship matrices. The $\Gamma_{148}$ matrix was tested in the ssGTBLUP approach and compared with UPG based ssGTBLUP. Results showed a slight improvement in prediction reliability and overprediction in the MF model over the UPG model.

## Data Availability

The data analyzed in this study was obtained from Finncattle Foundation (Finland), Finnish Breeder Association (FABA, Finland), Swedish Cattle Farmers Association (Växa), Landbrug and Fødevarer F.m.b.A (L and F), Nordic Cattle Genetic Evaluation (NAV, Denmark), Viking Genetics (Denmark), and corresponding farmers. Requests to access these datasets should be directed to the Director of Nordic Cattle Genetic Evaluation, Gert P. Aamand, gap@lf.dk.

## Appendix 1: Representation of the Φ_pre_ matrix used in formula ${\left({Ф}_{pre}^{\prime }{Ф}_{pre}\right)}^{-1}{{Ф}_{pre}^{\prime }\Gamma_{pre}Ф}_{pre}\;{\left({Ф}_{pre}^{\prime }{Ф}_{pre}\right)}^{-1}$ to compute the **K** matrix.

$$\begin{array}{c} Birth\;Year\\ 1970\\ 1980\\ 1990\\ 2000\\ 2010\\ 2021\\ 1970\\ 1980\\ 1990\\ 2000\\ 2010\\ 2021\\ 1980\\ 1990\\ 2000\\ 2010\\ 2021\\ 2000\\ 2021\\ 2000\\ 2021\\ 1990\\ 2000\\ 2021\\ 2000\\ 1960\\ 1980\\ 2000\\ 2020 \end{array}\left[\begin{array}{ccccccccc} \begin{array}{c} {STY}^{1}\\ -0.437\\ -0.155\\ 0.127\\ 0.409\\ 0.690\\ 0.972\\ -0.437\\ -0.155\\ 0.127\\ 0.409\\ 0.690\\ 0.972\\ -0.155\\ 0.127\\ 0.409\\ 0.690\\ 0.972\\ 0.409\\ 0.972\\ 0.409\\ 0.972\\ 0.127\\ 0.409\\ 0.972\\ 0.409\\ -0.718\\ -0.155\\ 0.409 \end{array} & \begin{array}{c} FIN\\ 1\\ 1\\ 1\\ 1\\ 1\\ 1\\ 0\\ 0\\ 0\\ 0\\ 0\\ 0\\ 0\\ 0\\ 0\\ 0\\ 0\\ 0\\ 0\\ 0\\ 0\\ 0\\ 0\\ 0\\ 0\\ 0\\ 0\\ 0 \end{array} & \begin{array}{c} SWE\\ 0\\ 0\\ 0\\ 0\\ 0\\ 0\\ 1\\ 1\\ 1\\ 1\\ 1\\ 1\\ 0\\ 0\\ 0\\ 0\\ 0\\ 0\\ 0\\ 0\\ 0\\ 0\\ 0\\ 0\\ 0\\ 0\\ 0\\ 0 \end{array} & \begin{array}{c} DNK\\ 0\\ 0\\ 0\\ 0\\ 0\\ 0\\ 0\\ 0\\ 0\\ 0\\ 0\\ 0\\ 1\\ 1\\ 1\\ 1\\ 1\\ 0\\ 0\\ 0\\ 0\\ 0\\ 0\\ 0\\ 0\\ 0\\ 0\\ 0 \end{array} & \begin{array}{c} NOR\\ 0\\ 0\\ 0\\ 0\\ 0\\ 0\\ 0\\ 0\\ 0\\ 0\\ 0\\ 0\\ 0\\ 0\\ 0\\ 0\\ 0\\ 1\\ 1\\ 0\\ 0\\ 0\\ 0\\ 0\\ 0\\ 0\\ 0\\ 0 \end{array} & \begin{array}{c} RDC\\ 0\\ 0\\ 0\\ 0\\ 0\\ 0\\ 0\\ 0\\ 0\\ 0\\ 0\\ 0\\ 0\\ 0\\ 0\\ 0\\ 0\\ 0\\ 0\\ 1\\ 1\\ 0\\ 0\\ 0\\ 0\\ 0\\ 0\\ 0 \end{array} & \begin{array}{c} FIC\\ 0\\ 0\\ 0\\ 0\\ 0\\ 0\\ 0\\ 0\\ 0\\ 0\\ 0\\ 0\\ 0\\ 0\\ 0\\ 0\\ 0\\ 0\\ 0\\ 0\\ 0\\ 1\\ 1\\ 1\\ 0\\ 0\\ 0\\ 0 \end{array} & \begin{array}{c} OTHER\\ 0\\ 0\\ 0\\ 0\\ 0\\ 0\\ 0\\ 0\\ 0\\ 0\\ 0\\ 0\\ 0\\ 0\\ 0\\ 0\\ 0\\ 0\\ 0\\ 0\\ 0\\ 0\\ 0\\ 0\\ 1\\ 0\\ 0\\ 0 \end{array} & \begin{array}{c} HOL\\ 0\\ 0\\ 0\\ 0\\ 0\\ 0\\ 0\\ 0\\ 0\\ 0\\ 0\\ 0\\ 0\\ 0\\ 0\\ 0\\ 0\\ 0\\ 0\\ 0\\ 0\\ 0\\ 0\\ 0\\ 0\\ 1\\ 1\\ 1 \end{array}\\ 0.972 & 0 & 0 & 0 & 0 & 0 & 0 & 0 & 1 \end{array}\right]$$

^1^ STY - birth year of the group standardized as $\frac{2\left({year}_{MF}-{year}_{\min}\right)}{{year}_{\max}-{year}_{\min}}-1$ , where *year*_MF_ is the year of the MF, *year*_*min*_ = 1950 and *year*_*max*_ = 2021.
